# Differences in Gaze Behavior Between Male and Female Elite Handball Goalkeepers During Penalty Throws

**DOI:** 10.3390/brainsci15030312

**Published:** 2025-03-15

**Authors:** Wojciech Jedziniak, Krystian Panek, Piotr Lesiakowski, Beata Florkiewicz, Teresa Zwierko

**Affiliations:** 1 Institute of Physical Culture Sciences, University of Szczecin, str. Piastów 40b, 71-065 Szczecin, Poland; wojciech.jedziniak@usz.edu.pl (W.J.); beata.florkiewicz@usz.edu.pl (B.F.); 2 Department of Physical Culture and Health, Institute of Physical Culture Sciences, Students of Sport Diagnostics Faculty, University of Szczecin, str. Piastów 40b, 71-065 Szczecin, Poland; krystianpanek99@wp.pl; 3 Department of Physical Education and Sport, Pomeranian Medical University, str. Dunikowskiego 6a, 70-123 Szczecin, Poland; piotr.lesiakowski@pum.edu.pl

**Keywords:** gaze strategy, quiet eye, mobile eye-tracker, sport expertise, sex differences

## Abstract

Background: Recent research suggests that an athlete’s gaze behavior plays a significant role in expert sport performance. However, there is a lack of studies investigating sex differences in gaze behavior during technical and tactical actions. Objectives: Therefore, the purpose of this study was to analyze the eye movements of elite female and male handball goalkeepers during penalty throws. Methods: In total, 40 handball goalkeepers participated in the study (female: *n* = 20; male: *n* = 20). Eye movements were recorded during a series of five penalty throws in real-time conditions. The number of fixations and dwell time, including quiet eye, for selected areas of interest were recorded using a mobile eye-tracking system. Results: Significant differences were found in quiet-eye duration between effective and ineffective goalkeeper interventions (females: mean difference (MD) = 92.26; *p* = 0.005; males: MD = 122.83; *p* < 0.001). Significant differences in gaze behavior between female and male handball goalkeepers were observed, specifically in the number of fixations and fixation duration on the selected areas of interest (AOIs). Male goalkeepers primarily observed the throwing upper arm AOI, the throwing forearm (MD = 15.522; *p* < 0.001), the throwing arm AOI (MD = 6.83; *p* < 0.001), and the ball (MD = 7.459; z = 3.47; *p* < 0.001), whereas female goalkeepers mainly observed the torso AOI (MD = 14.264; *p* < 0.001) and the head AOI (MD = 11.91; *p* < 0.001) of the throwing player. Conclusions: The results suggest that female goalkeepers’ gaze behavior is based on a relatively constant observation of body areas to recall task-specific information from memory, whilst male goalkeepers mainly observe moving objects in spatio-temporal areas. From a practical perspective, these results can be used to develop perceptual training programs tailored to athletes’ sex.

## 1. Introduction

High levels of perceptual–cognitive skills, such as visual search, the identification and processing of environmental information, effective selection of external stimuli, anticipation, and decision-making, determine elite athletes’ performance [1,2]. The results of experimental studies have shown that players with greater sports experience have the ability to effectively gather relevant information from areas of their opponents’ bodies [3] and from the technical and tactical structure of the game [4], which consequently allows for more accurate predictions of opponents’ and teammates’ behavior during rapidly changing game situations [5].

Therefore, it has been suggested that specific gaze behavior is an important determinant of players’ perceptual–cognitive competence [6]. Gaze behavior is the purposeful use of the visual system to focus on areas of interest (AOIs) in a relatively fixed sequence, recognize the movements of an opponent, and make an appropriate response [7]. Recent findings have shown that gaze behavior, in terms of oculomotor functions (i.e., fixation points, fixation duration, and dwell time), depends on the characteristics of the sport and the difficulty of the motor task [8]. Moreover, the gaze behavior adopted significantly differentiates players with a high level of sport expertise from novice players in terms of the overall number of fixations and fixation duration [9,10,11]. Expert–novice differences were also reported with regard to different AOIs being used during offensive and defensive motor tasks [12,13]. In defensive actions in various sport disciplines, gaze behavior is dominated by picking up relevant cues from areas of the opponent’s body that indicate how the motor task will be performed [14,15,16]. A previous study of gaze strategies in handball showed that experienced goalkeepers made more fixations on the throwing arm of the penalty thrower, whereas novice goalkeepers made more fixations on the head of the penalty thrower [17]. Moreover, it has been indicated that observation of the throwing arm of the penalty thrower is crucial in assessing the type of throw that will be used [18].

Studies examining the effectiveness of visual strategies in athletes emphasize the impact of “quiet eye” on the accuracy and efficiency of motor performance [19,20,21]. The paradigm of quiet eye, as defined by Vickers [22], refers to the duration of the final fixation lasting longer than 100 ms and occurring within three degrees of the visual field, immediately prior to the initiation of a motor task. This phenomenon is associated with optimal focus and reduced cognitive interference, allowing for better preparation and execution of motor actions. Research consistently shows that elite athletes, in comparison to less skilled performers, demonstrate significantly longer durations of visual stability [23,24]. This prolonged fixation is believed to facilitate better anticipation, decision-making, and motor planning, which are critical for performance in dynamic sports environments.

Although the relationship between gaze behavior and athletes’ performance has been well documented, there is a lack of studies investigating differences between female and male gaze strategies during technical and tactical actions. So far, most studies have analyzed gaze behavior in athletes of the same gender [25,26]. However, research does indicate some sex differences in visuospatial planning (i.e., men tend to be better at tasks that require spatial orientation and navigation, whilst women show better results for object localization) [27,28]. Moreover, in visuospatial planning tasks, men frequently change the planned pattern of activities, whilst women prefer to use a consistent approach. Men, compared to women, have shown a higher level of achievement in behavioral actions, with better oculomotor function efficiency found [29]. Differences between women and men in tasks requiring memory have also been found [30], with women achieving better results compared to men [31]. In general terms, it is thought that highly qualified players possess a superior ability to encode and recall information from memory more rapidly than less qualified players [32]. Thus, differences between the sexes may have an impact on the gaze strategies used when undertaking motor tasks.

The aim of the present research was to evaluate the gaze strategies of female and male handball goalkeepers during a handball penalty throw. The penalty throw is a fundamental part of handball, occurring in various match situations and often determining the final outcome. Due to its importance, goalkeepers must refine their perceptual and decision-making skills to maximize their effectiveness in these scenarios. The standardized nature of penalty throws provides a controlled environment for analyzing gaze behavior, offering valuable insights into the visual strategies used in high-pressure situations. Previous non-sport-specific research suggests that differences may exist between men and women in planning and executing motor activities. Therefore, goalkeepers might adopt different gaze strategies, particularly regarding the number of fixations and fixation duration on specific AOIs [31]. It is expected that female goalkeepers will demonstrate a higher number of fixations on a greater number of AOIs compared to male goalkeepers during penalty throws [33]. From a practical point of view, the findings of this study may provide valuable insights for coaches, trainers, and specialists involved in the preparation of handball goalkeepers. Understanding the specific visual strategies employed by goalkeepers allows for the development of more effective training programs that can enhance intervention efficiency.

## 2. Materials and Methods

### 2.1. Participants

An a priori statistical power analysis was performed using G*Power 3.1 software. Based on the a priori analysis, for *t*-tests, we adopted a power of 0.8, an alpha level of 0.05, and an effect size of 0.85. The power analysis identified that a sample size of 40 participants was sufficient for this study. An observational, comparative study design with purposive sampling was employed in accordance with established research procedures, using a mobile eye-tracking system in real-world sports conditions [9]. A total of 40 highly qualified female and male handball goalkeepers were recruited. Female goalkeepers (F; *n* = 20) had a mean age of 23.30 years (SD = 6.52 years) and mean sport experience of 12.34 years (SD = 4.71 years). Male goalkeepers (M; *n* = 20) had a mean age of 25.85 years (SD = 4.05 years) and mean sport experience of 12.82 years (SD = 4.89 years). Both female and male goalkeepers were players for clubs at the highest level of competition in Poland (Superliga), with several goalkeepers also being members the national team of Poland (FG = 4; MG = 3), corresponding to Tier 4 (Elite/International Level) of McKay’s participant classification framework [34]. The inclusion criteria for goalkeepers in this study were as follows: (1) a minimum of 10 years of competitive experience as a handball goalkeeper, (2) nomination to the central goalkeeper training program of the national team in the year of the study, and (3) active participation in the highest level of domestic competition (Superliga) during the study period. These criteria ensured that all participants had a high level of expertise and were trained under standardized conditions. This study was approved by the local bioethical committee (No. 11/KB/V/2017).

### 2.2. Gaze Data

**Figure 1:**
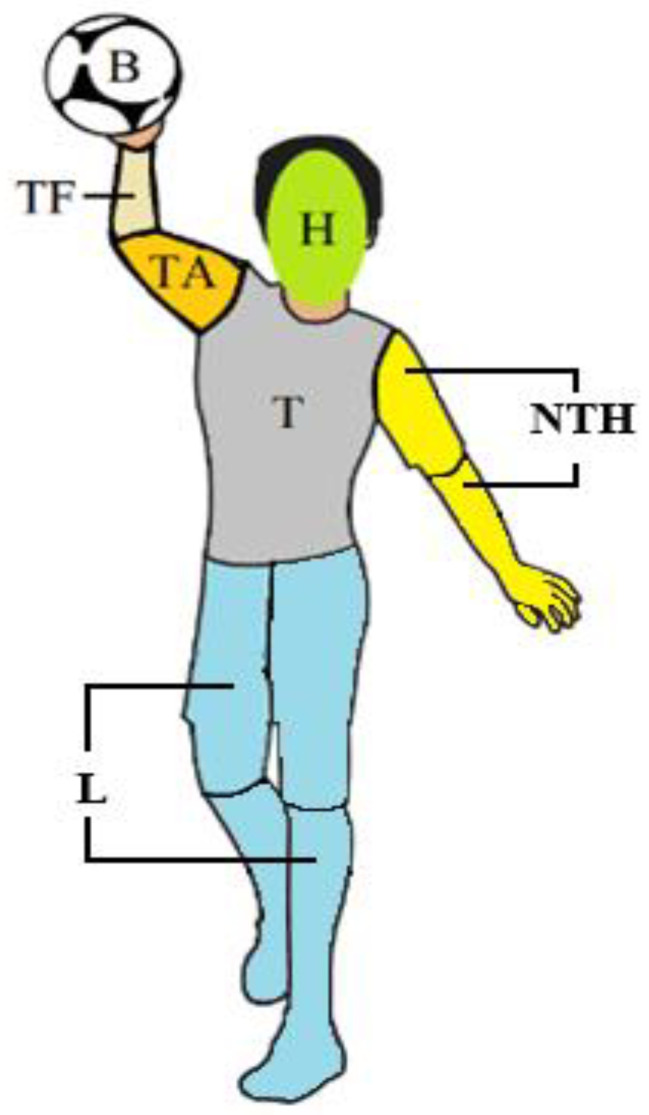
Reference image of a handball penalty thrower with selected areas of interest (AOI): head (H), torso (T), throwing arm (TA), throwing forearm (TF), ball (B), non-throwing hand (NTH), legs (L).

A mobile eye-tracking system (ET) consisting of glasses and an image-recording controller (SMI ETG 2w, SensoMotoric Instruments GmbH, Teltow, Germany) was used to assess eye movements when defending a handball penalty throw. The system allows the analysis of eye movements in real-time conditions. The ET glasses had an HD scene camera (1280 × 960 px; 24 fps), automatic parallax correction, and a frequency of 60 Hz. Participants wore an eye tracker connected to a SAMSUNG Galaxy S4 smartphone (Samsung Electronics, Suwon, South Korea), which recorded gaze data throughout the task. The smartphone was securely placed in a waist pouch positioned on the participant’s back to ensure unrestricted movement and minimize interference with performance. The data were analyzed frame by frame using BeGaze 3.5 software. The results were analyzed using a Lenovo X230 computer with Intel^®^ core™ i7-3520 M, 2.90 GHz, and 4 GB RAM. There were 200 penalty throws in total. The eye movements of each participant were tracked using a reference image of a penalty thrower (Figure 1) with the following AOIs: head (H), torso (T), throwing arm (TA), throwing forearm (TF), ball (B), non-throwing hand (including arm and forearm) (NTH), legs (L) (including thigh and shank of right and left legs), and other (O) (where the fixation could not be counted for any previous AOI). The data collected allowed for the following variables to be calculated: total observed AOIs [%], percentage distribution of fixation on AOIs (%), percentage of viewing time on AOIs [%], total number of fixations [n], quiet-eye duration [ms], and average fixation duration [ms].

### 2.3. Procedure

The goalkeepers were instructed to defend a penalty throw that was taken in accordance with the rules of the International Handball Federation (IHF). According to the IHF rules, the goalkeeper can defend the ball with the entire surface of their body, with the only limitation of the goalkeeper’s actions being the 4 m distance of the goalkeeper from the goal line. As the penalty throw was performed in real-time conditions, the IHF rules concerning the throwing player were also taken into account. The throwing player is placed in front of the 7 m line, has 3 s to throw once the referee’s whistle sounds, and must keep one foot in contact with the ground. During the study, no breaches of the rules by the goalkeepers or throwing players were observed. The tests were preceded by a 15 min standardized warm-up. The single-point method was used to calibrate the ET glasses for each participant. Whilst wearing the ET glasses, participants stood at distance of 1 m from a wall and looked at a 2 cm diameter circle located at a height of 180 cm on the wall for 10–15 s until the calibration was complete. Following calibration, a familiarization period of 5 min was provided, where goalkeepers performed handball techniques (passes, grips, throws) whilst wearing the ET glasses.

To simulate match conditions as closely as possible, penalty throws were taken by two highly qualified female handball players for the female goalkeepers and two highly qualified male handball players for the male goalkeepers. The throwers were selected based on their sporting experience, which corresponded to the level of the analyzed goalkeepers [35,36]. Regarding methodological validity, throwers were only substituted if necessary [37]. A one-minute break was applied between consecutive penalty throw interventions. Taking into account the IHF handball rules, different sizes of ball were used (size 3 for men, size 2 for women). Based on the results by Loffing et al. [38], who observed lower defensive effectiveness in handball goalkeepers when facing penalty throws by left-handed players, all penalties in this study were taken by right-handed players.

### 2.4. Warm-Up Protocol

The tests were preceded by a 15 min standardized warm-up. Participants performed the same warm-up routine, consisting of specific components, with the total duration of the warm-up being approximately 15 min, as follows: (A) aerobic phase—exercises comprising jogging, joint mobility exercises (e.g., hip mobility, etc.), and dynamic stretching (~5′); (B) neuromuscular phase, including strengthening (e.g., half-squat) and balance exercises (~2′); (C) goalkeeper-specific phase. This phase included (i) footwork and reaction speed, where the goalkeeper practices lateral and forward–backward movements, ensuring agility in the goal area. Reaction drills follow in signaled directions, prompting quick responses. The segment ends with simulation exercises—practicing low and high saves without a ball, reinforcing defensive mechanics (~2′). (ii) Catching drills were also carried out (~6′), focusing on single-handed catches at different angles and bouncing the ball off a wall to improve handling. Reaction exercises follow, where the player throws balls at different speeds and heights, requiring rapid adjustments. The warm-up concludes with light-intensity shot-saving drills, ensuring the goalkeeper is fully prepared.

### 2.5. Statistical Analysis

The distribution of variables was analyzed using the Shapiro–Wilk test. The results indicated that the variables followed a non-parametric distribution (*p* > 0.05). The Mann–Whitney U test was applied to examine significant differences between male and female goalkeepers and to compare variables within each group based on effective and ineffective goalkeeper interventions. To accurately present the obtained results in descriptive statistics, the mean and standard deviation, mean rank for the groups, and the U and Z coefficients of the Mann–Whitney U test were used. The rank biserial correlation coefficient (rb) was used to assess the effect size, with the following interpretations: weak effect = 0.10, moderate effect = 0.30, strong effect = 0.50 [39].

## 3. Results

### 3.1. Number of Fixations on AOIs

The analysis revealed significant differences between male and female goalkeepers in the number of fixations made in specific areas of interest (AOIs) (Table 1). It was observed that male goalkeepers performed a higher number of fixations compared to female goalkeepers during a penalty throw in handball (MD = 0.84; Z = 1.12; *p* < 0.001). However, no significant difference was found in the total number of observed areas of interest (AOIs) (MD = 2.42; Z = 3.91; *p* = 0.259). The analysis also indicated that different areas of interest (AOIs) were crucial for male and female goalkeepers during penalty throw interventions. Female goalkeepers made more frequent observations of the head AOI (MD = 11.91; Z = −4.63; *p* < 0.001) and torso AOI (MD = 14.264; Z = −6.44; *p* < 0.001) compared to male goalkeepers. On the other hand, the visual strategy of male goalkeepers was characterized by a greater number of fixations on the throwing forearm AOI (MD = 15.522; Z = 5.93; *p* < 0.001), throwing arm (MD = 6.83; Z = 2.41; *p* < 0.001), and ball AOI (MD = 7.459; Z = 3.47; *p* < 0.001) compared to female goalkeepers.

### 3.2. Fixation Duration on AOIs

The analysis revealed significant differences between male and female goalkeepers in the percentage of viewing time of specific areas of interest (AOIs) (Table 2). No significant differences were found in the duration of quiet eye between female and male goalkeepers (MD = 22.41; Z = 0.261; *p* = 0.795). However, female goalkeepers demonstrated a significantly longer average fixation duration compared to male goalkeepers (MD = 49.85; Z = −4.01; *p* < 0.001). Moreover, the analysis showed that different areas of interest (AOIs) were significant in terms of observation duration during a penalty throw, depending on sex. Female goalkeepers spent more time observing the head AOI (MD = 15.77; Z = −5.82; *p* < 0.001), torso AOI (MD = 13.361; Z = −5.65; *p* < 0.001), and legs AOI (MD = 3.08; Z = −1.72; *p* = 0.031) compared to male goalkeepers. The visual strategy of male goalkeepers was characterized by longer fixation durations on the throwing forearm AOI (MD = 16.215; Z = 5.94; *p* < 0.001), throwing arm (MD = 8.17; Z = 3.16; *p* = 0.001), and ball AOI (MD = 8.14; Z = 3.57; *p* < 0.001) compared to female goalkeepers.

### 3.3. Number of Fixations and Effectiveness of Interventions

The aim of the analysis was to identify differences in fixation parameters, taking into account the effectiveness of interventions separately for female and male goalkeepers (Table 3).

In the group of female goalkeepers, the analysis revealed significant differences between effective and ineffective interventions in relation to the number of fixations directed toward the torso AOI. During effective interventions, women significantly more frequently observed the torso AOI (MD = 13.033; Z = 3.72; *p* < 0.001) compared to ineffective interventions. Furthermore, in effective interventions, women performed fewer overall fixations compared to ineffective ones (MD = −0.773; Z = 2.08; *p* = 0.031). A similar pattern was observed for the number of observed areas of interest (AOIs observed) and the ball AOI—during effective interventions, women focused their gaze on fewer AOIs (MD = −9.54; Z = 3.09; *p* = 0.001) and ball AOIs (MD = −6.361; Z = 2.06; *p* = 0.021) compared to ineffective interventions.

For male goalkeepers, a higher number of fixations on the throwing forearm AOI were observed during effective interventions compared to ineffective ones (MD = 18.0; Z = 4.56; *p* < 0.001). Additionally, during effective interventions, men directed their attention less frequently to the head AOI compared to ineffective interventions (MD = −10.37; Z = 3.34; *p* < 0.001).

### 3.4. Fixation Duration on AOIs and Effectiveness of Interventions

The analysis of the results on differences in percentage viewing time, considering the effectiveness of interventions separately for female and male goalkeepers (Table 4), revealed significant differences in quiet-eye duration for both groups. Female and male goalkeepers exhibited longer quiet eye during effective interventions compared to ineffective ones (females; MD = 92.26; Z = 2.77; *p* = 0.005; males; MD = 122.83; Z = 4.76; *p* < 0.001). In the group of female goalkeepers, a longer average fixation duration was observed during effective interventions compared to ineffective ones (MD = 54.18; Z = −2.61; *p* = 0.009). Furthermore, a significantly longer fixation duration was noted on the torso AOI during effective interventions compared to ineffective ones (MD = 14.23; Z = 4.37; *p* < 0.001). Conversely, during ineffective interventions, female goalkeepers spent significantly more time observing the ball AOI (MD = 6.55; Z = 2.01; *p* = 0.024) and the throwing forearm AOI (MD = 7.63; Z = 3.19; *p* < 0.001). In the group of male goalkeepers, effective interventions were associated with longer observation times on the throwing forearm AOI compared to ineffective interventions (MD = 19.27; Z = 4.77; *p* < 0.001). Additionally, a shorter observation time was recorded for the head AOI during effective interventions compared to ineffective ones (MD = 12.24; Z = −4.08; *p* < 0.001).

## 4. Discussion

The main findings are as follows: (1) Female and male handball goalkeepers exhibit different gaze strategies. Female goalkeepers focus their visual attention primarily on the head and torso of the throwing player, whereas male goalkeepers concentrate on the spatio-temporal area, specifically the throwing arm and forearm. (2) The sex of the goalkeeper influences gaze behavior, particularly in terms of the total number of fixations on AOIs during penalty throw execution. (3) No differences in quiet-eye duration were observed between female and male goalkeepers. However, significant differences in quiet-eye duration were identified within both groups when comparing effective and ineffective interventions.

In terms of quiet eye, significant differences were observed between effective and ineffective interventions among the goalkeepers studied. The present findings align with previous studies [19,20,21] indicating that longer quiet-eye duration improves the effectiveness of specific motor actions. However, no significant differences in quiet-eye duration were found between female and male goalkeepers. From a neuro-behavioral perspective, quiet-eye duration is associated with attentional control processes [40], with some evidence suggesting a slight advantage for women over men [41]. At the same time, studies on visual strategies in specific motor tasks suggest that quiet-eye duration varies depending on the task characteristics and the impact of fatigue [9,42]. A penalty throw in handball is a task performed under great time pressure. It is possible that participation in long-term training processes enables both male and female goalkeepers to adjust their quiet-eye duration, potentially eliminating differences between the sexes in this regard.

The results of our study are in line with previous reports on gaze strategies used for specific motor tasks in various sports disciplines [14,15,16]. The male goalkeepers’ results are consistent with the previous findings of Rivilla-Garcia et al. [17], who showed that highly qualified handball goalkeepers observed the throwing arm of the throwing player and the ball significantly more often during handball penalty throws compared to novice goalkeepers. Research has indicated that looking at the throwing hand allows the trajectory of the ball [17] and type of throw [18] to be determined, and based on our findings, positively influences the male handball goalkeepers’ effectiveness against penalty throws. In contrast, female goalkeepers predominantly fixated on the torso area of the throwing player during penalty throws. Observing the torso of the throwing player may provide female handball goalkeepers with critical predictive information regarding both the direction and timing of an incoming penalty throw. This suggests that goalkeepers rely not only on the motion of the throwing arm, but also on the kinematics of the upper body to anticipate the opponent’s actions. Additionally, fixating on the torso and head region may support the adoption of a gaze strategy that leverages peripheral vision, known as the visual pivot [43]. The visual pivot refers to final fixations that can be adjusted through smooth-pursuit eye movements, allowing for more efficient tracking of the thrower’s movements while maintaining awareness of key peripheral visual cues. This strategy enables goalkeepers to rapidly analyze their opponent’s actions and quickly return to crucial areas of interest (AOIs) [44]. By optimizing gaze behavior, the visual pivot not only enhances reaction speed, but also improves the processing of peripheral dynamic cues, such as the movement trajectory of the throwing arm and the shifting posture of the upper body. This refined visual processing mechanism contributes to more effective decision-making in penalty defense situations [2]. Moreover, female goalkeepers during handball penalty throws made more fixations on the thrower’s head. It is possible that this is related to the frontal set-up of the goalkeeper and throwing player. A previous study from Brandow and Witte [13] also indicated that experienced karate fighters observed the opponent’s head more frequently, as this provides information about the execution of attacks.

Our findings confirmed a difference between female and male goalkeepers in the total number of fixations on AOIs, with male goalkeepers making more fixations than female goalkeepers, which contradicts our initial assumption. Previous studies have shown that women, compared to men, tend to use a greater number of fixations and, consequently, a longer planning time in navigation tasks [27] and picture-viewing tasks [33]. In our study, it is plausible that the short duration of the motor task (i.e., penalty throw) influenced the gaze behavior. Additionally, male goalkeepers may have made a greater number of fixations due to more frequent adjustments in action planning. This explanation appears reasonable in light of the findings by Cazzato et al. [29], who suggest that, in visuospatial planning tasks, men rely more on a spatially based problem-solving approach, whereas women focus more on executing the plan.

Moreover, for both female and male handball goalkeepers, different areas of the throwing player’s body play a crucial role in successfully defending a penalty throw. The observed differences in gaze strategies suggest that male goalkeepers primarily rely on dynamic spatial cues, such as the movement of the throwing arm and ball, to predict shot direction. This aligns with previous research indicating that men tend to excel in spatial orientation and dynamic tracking tasks. In contrast, female goalkeepers appear to adopt a more stable reference framework, focusing on the torso and head. This may indicate a greater reliance on memory-based processing and object recognition—cognitive strategies in which women often outperform men. These findings are consistent with the results of Mueller et al. [31], who demonstrated that men tend to analyze real-time spatial relationships in spatial planning tasks, whereas women prefer strategies based on recalling prior experiences.

Furthermore, it was shown that women achieved better results compared to men in a memory task, with women able to encode more detail about the task structure [30]. It is possible that based on the observation of the torso and head of the throwing player during penalty throws, female goalkeepers recalled from memory the structure of the task and chose the direction and method of their save attempt without focusing as much on the throwing hand. At the same time, from a practical point of view, it seems that schematic decision-making is easier to “read” for opponents.

Differences in the structure of female and male brains may provide some explanation for the differences observed in the gaze strategies of handball goalkeepers. Gur et al. [45] indicated that, in men, a larger layer of white matter in the right hemisphere of the brain allows for better performance in tasks requiring spatial orientation, whilst women, with a thicker layer of gray matter in the left hemisphere, obtain better results in tasks requiring the verbal assessment of situations. In general, it has been assumed that men have an advantage over women when processing visuospatial information [46]. A study by Vogel et al. [47] showed a difference in the activation of cerebral hemispheres. In spatial orientation tasks, increased activity in the right hemisphere of the brain was observed in men, whilst women activated both hemispheres. The authors argued that such action could be the basis for more effective problem-solving in spatial orientation tasks. On the other hand, in visual reflexive attention tasks with event-related potentials (ERPs), it was revealed that women exhibited greater ERP component amplitudes. This may result from the fact that women’s need for visuospatial attention arises earlier than in men, which leads to high activation patterns with increased amplitudes in the parietal and frontal brain regions [41]. Moreover, in memory tasks, Hughdal et al. [48] observed a difference in activity in Brodmann area 7, which is responsible for associations between visual stimulation and motor activities. The authors suggested that different levels of activation in the frontal lobes might be the result of undertaking different strategies when undertaking visuospatial tasks. Taken together, the results of this study corroborate the differences in the gaze strategies of women and men during motor tasks.

### Limitations and Future Research

Whilst our findings provide novel insights into differences between female and male goalkeepers’ gaze strategies during handball penalty throws, this study does have limitations. Firstly, despite collecting data under real-time conditions, it was not possible to replicate the influence of factors that occur during handball matches, such as emotions, pressure, and fatigue. These factors may influence the gaze strategies used [42]. Second, the fact that there were different performers of the penalty throw (male and female) may decrease the strength and validity of our outcomes. However, a previous report by Van Den Tillaar and Cabri [49] indicated that ball release velocity was higher in men compared to women, but kinematic analysis showed that both genders use the same throwing technique during handball penalty throws. Moreover, the handball rules (IHF) specify different ball sizes for women (size 2) and men (size 3), and direct confrontation between male and female players does not occur in competitive handball.

Additionally, a potential limitation of this study is that each goalkeeper faced penalty throws from the same attacker, which may not fully reflect the variability present in real match conditions. In actual game situations, goalkeepers must adapt to the different shooting styles, techniques, and decision-making patterns of multiple opponents. Future research should consider incorporating penalty throws from multiple attackers to examine whether goalkeepers adjust their gaze strategies depending on the characteristics of different shooters. This approach could provide a more comprehensive understanding of perceptual–cognitive adaptations in goalkeeping.

Future research should also limit the goalkeeper’s output, as positioning may alter gaze strategy. Additionally, it is necessary to assess oculomotor function in other types of throws, given the possibility of different gaze strategies occurring under changing environmental conditions. This information seems beneficial for understanding the extent of goalkeepers’ perceptual awareness in directing visual attention to specific areas of interest during defensive actions.

A further limitation of this study is that it does not consider the role of social attention in gaze behavior during penalty throws. Social cues, such as facial expressions and body posture, influence attentional processes [50] and may have contributed to the increased focus on the head and torso observed in female goalkeepers. Future research should explore how goalkeepers process and respond to these cues to better understand their impact on decision-making in defensive actions.

## 5. Practical Applications

The findings of this study have important practical implications for sports training, particularly for goalkeepers in penalty situations. From a training perspective, understanding differences in gaze strategies can help coaches tailor training programs to optimize visual search patterns and improve decision-making under time constraints. For instance, male goalkeepers may benefit from training methods that enhance efficient fixation distribution and quick adaptation to dynamic task situations. On the other hand, female goalkeepers might focus on strategies that refine fixation efficiency and reaction times. Additionally, our findings can be used to develop perceptual training programs and support young players in improving their technical and tactical preparation for penalty throws [51]. A broader understanding of gaze behavior in handball may enhance training effectiveness and introduce additional criteria for talent identification, as previously proposed in football [52].

## 6. Conclusions

Our study highlighted significant differences in gaze strategies between female and male handball goalkeepers during penalty throws, primarily in the number of fixations and fixation duration. Specifically, female goalkeepers tend to focus more on the opponent’s head and torso, whereas male goalkeepers predominantly direct their attention toward the throwing arm, forearm, and ball.

These differences suggest distinct perceptual–cognitive strategies based on gender, which may influence goalkeepers’ ability to anticipate and effectively defend against penalty throws. Additionally, our findings indicate that a longer quiet-eye duration positively impacts successful interventions for both male and female goalkeepers.

This study provides valuable insight into gaze strategies at the elite level, contributing to the understanding of perceptual–cognitive mechanisms in high-performance handball. However, as our research focused on elite athletes, future studies should examine whether similar patterns occur in goalkeepers with varying levels of experience.

## Figures and Tables

**Table 1:** Number of fixations on selected areas of interest (AOIs; [%]) made by handball goalkeepers during penalty throws.

AOI [%]	Group	Mean	SD	Mean Rank	*U*	*Z*	*r_b_*	*p*
TFA	F	7.074	11.000	76.205	2570.50	5.93	−0.486	***
	M	22.626	19.755	124.795				
H	F	29.717	18.317	119.465	3103.50	−4.63	0.379	***
	M	17.811	14.497	81.535				
B	F	9.158	13.571	86.265	3576.50	3.47	−0.285	***
	M	16.617	15.627	114.735				
TA	F	18.064	14.564	90.620	4012.00	2.41	−0.198	**
	M	24.447	17.597	110.380				
O	F	3.530	8.490	99.775	4927.00	0.17	0.014	ns
	M	3.254	6.809	101.225				
L	F	6.299	9.627	107.320	4318.00	−1.66	0.136	ns
	M	3.352	6.342	93.680				
T	F	26.158	17.070	126.865	2363.50	−6.44	0.527	***
	M	11.894	12.642	74.135				
Fixation [n]	F	6.170	1.753	84.500	3400.00	1.12	−0.321	***
	M	7.010	1.703	116.500				
AOI observed	F	54.429	14.314	95.880	4538.00	3.91	−0.092	ns
	M	56.857	13.466	105.120				

AOI—area of interest; F—female goalkeepers; M—male goalkeepers; TFA—throwing forearm; H—head; B—ball; TA—throwing arm; O—other; L—legs; T—torso; *r_b_*—rank biserial correlation; *U*—Mann–Whitney U statistic; *Z*—z score; ns—not significant; ** *p* < 0.01; *** *p* < 0.001. For a more detailed breakdown of the results, see Table S1 in the Supplementary Materials.

**Table 2:** Percentage of viewing time on selected areas of interest (AOIs) by handball goalkeepers during penalty throws.

AOI	Group	Mean	SD	Mean Rank	*U*	*Z*	*r_b_*	*p*
Qe [ms]	F	371.050	198.931	99.430	4893.00	0.261	−0.021	ns
	M	348.640	143.242	101.570				
Avg fx [ms]	F	395.354	101.303	116.940	3356.00	−4.01	0.329	***
	M	345.503	83.181	84.060				
TFA	F	6.555	10.239	76.145	2564.50	5.94	−0.487	***
	M	22.770	21.093	124.855				
H	F	33.614	19.325	124.355	2614.50	−5.82	0.477	***
	M	17.828	15.083	76.645				
B	F	8.150	12.813	85.870	3537.00	3.57	−0.293	***
	M	16.299	16.226	115.130				
TA	F	16.277	14.120	87.540	3704.00	3.16	−0.259	**
	M	24.450	18.167	113.460				
O	F	3.174	7.641	99.590	4909.00	0.22	−0.018	ns
	M	2.864	6.415	101.410				
L	F	6.238	9.654	107.555	4294.50	−1.72	0.141	*
	M	3.158	6.202	93.445				
T	F	25.992	16.944	123.630	2687.00	−5.65	0.463	***
	M	12.631	13.647	77.370				

AOI—area of interest; Qe—quiet-eye duration; Avg fx—average fixation duration; F—female goalkeepers; M—male goalkeepers; TFA—throwing forearm; H—head; B—ball; TA—throwing arm; O—other; L—legs; T—torso; *r_b_*—rank biserial correlation; *U*—Mann–Whitney U statistic; *Z*—z score; ns—not significant * *p* < 0.05; ** *p* < 0.01; *** *p* < 0.001.

**Table 3:** Analysis of number of fixations on selected areas of interest (AOIs; %), including goalkeepers’ effectiveness.

AOI	Group	Mean ± SD		Mean ± SD		Mean Rank		*U*	*Z*	*r_b_*	*p*
		Save		Goal		Save	Goal				
Fixation_[n]	F	5.776	1.598	6.549	1.826	44.316	56.441	946.50	2.08	−0.242	*
	M	6.878	1.589	7.137	1.811	48.551	52.373	1154.00	−0.65	−0.076	ns
AOI observed	F	49.563	13.709	59.104	13.407	41.327	59.314	800.00	3.09	−0.360	**
	M	55.102	13.678	58.543	13.172	47.265	53.608	1091.00	−1.08	−0.127	ns
TFA	F	3.679	7.607	10.335	12.723	42.837	57.863	874.00	2.58	−0.301	**
	M	31.810	20.303	13.801	14.658	64.010	37.520	1911.00	4.56	0.530	***
H	F	32.707	17.983	26.844	18.350	55.245	45.941	1482.00	−1.59	0.186	ns
	M	12.524	11.084	22.891	15.635	40.602	60.010	764.50	−3.34	−0.388	***
B	F	5.914	10.325	12.275	15.559	44.388	56.373	950.00	2.06	−0.240	*
	M	17.075	15.899	16.177	15.507	51.255	49.775	1286.50	0.25	0.030	ns
TA	F	15.327	14.369	20.693	14.401	44.898	55.882	975.00	1.88	0.220	ns
	M	23.157	18.102	25.685	17.184	48.061	52.843	1130.00	−0.82	−0.096	ns
O	F	3.950	8.280	3.126	8.750	51.786	49.265	1312.50	−0.43	0.050	ns
	M	2.978	7.096	3.519	6.581	49.020	51.922	1177.00	−0.49	−0.058	ns
L	F	5.618	9.474	6.953	9.822	48.622	52.304	1157.00	0.63	−0.074	ns
	M	2.250	5.683	4.411	6.804	46.469	54.373	1052.00	−1.35	−0.158	ns
T	F	32.805	20.317	19.772	9.786	61.541	39.892	1790.50	−3.72	0.433	***
	M	10.206	11.606	13.516	13.478	47.347	53.529	1095.00	−1.06	−0.124	ns

AOI—area of interest; F—female goalkeepers; M—male goalkeepers; TFA—throwing forearm; H—head; B—ball; TA—throwing arm; O—other; L—legs; T—torso; *r_b_*—rank biserial correlation; *U*—Mann–Whitney U statistic; *Z*—z score; ns—not significant; * *p* < 0.05; ** *p* < 0.01; *** *p* < 0.001.

**Table 4:** Percentage of viewing time on areas of interest (AOIs), including goalkeepers’ intervention effectiveness.

AOI	Group	Mean ± SD		Mean ± SD		Mean Rank		*U*	*Z*	*r_b_*	*p*
		Save		Goal		Save	Goal				
Qe [ms]	F	418.102	207.526	325.843	180.990	58.724	42.598	846.50	2.77	0.323	**
	M	411.571	130.865	288.176	128.745	64.602	36.951	558.50	4.76	0.553	***
Avg fx [ms]	F	422.988	109.792	368.803	85.322	58.245	43.059	870.00	−2.61	0.304	**
	M	349.624	80.049	341.544	86.692	52.602	48.480	1146.50	0.71	0.082	ns
TFA	F	2.659	5.767	10.298	12.103	41.041	59.588	786.00	3.19	−0.371	***
	M	32.599	21.209	13.327	16.231	64.633	36.922	557.00	4.77	0.554	***
H	F	34.713	18.120	32.559	20.540	51.571	49.471	1197.00	−0.35	0.042	ns
	M	11.196	10.255	24.200	16.278	38.388	62.137	656.00	−4.08	−0.475	***
B	F	4.809	8.182	11.359	15.471	44.531	56.235	957.00	2.01	−0.243	*
	M	17.267	17.207	15.369	15.339	51.837	49.216	1184.00	0.44	0.052	ns
TA	F	15.136	14.410	17.372	13.890	48.000	52.902	1127.00	0.84	−0.098	ns
	M	24.473	19.743	24.428	16.712	50.071	50.912	1228.50	−0.14	−0.017	ns
O	F	3.263	7.245	3.089	8.074	51.500	49.539	1200.50	−0.33	0.039	ns
	M	2.311	5.984	3.394	6.821	48.837	52.098	1168.00	−0.55	−0.065	ns
L	F	6.169	10.309	6.304	9.083	49.571	51.392	1204.00	0.31	−0.036	ns
	M	1.854	4.796	4.410	7.128	45.939	54.882	1026.00	−1.53	−0.179	ns
T	F	33.250	20.279	19.018	8.512	63.469	38.039	614.00	4.37	0.509	***
	M	10.300	11.684	14.871	15.075	46.316	54.520	1044.00	−1.41	−0.164	ns

AOI—area of interest; Qe—quiet-eye duration; Avg fx—average fixation duration; F—female goalkeepers; M—male goalkeepers; TFA—throwing forearm; H—head; B—ball; TA—throwing arm; O—other; L—legs; T—torso; *r_b_*—rank biserial correlation; *U*—Mann–Whitney U statistic; *Z*—z score; ns—not significant * *p* < 0.05; ** *p* < 0.01; ***p < 0.001.

## Data Availability

The original data presented in the study are openly available in RepOD at DOI: https://doi.org/10.18150/D1B2BE. The dataset can be accessed at the following link: https://repod.icm.edu.pl/dataset.xhtml?persistentId=doi%3A10.18150%2FD1B2BE (accessed on 7 March 2025).

## Supplementary Materials

The following supporting information can be downloaded at: https://www.mdpi.com/article/10.3390/brainsci15030312/s1, Table S1: Data on gaze behavior during handball penalty throw.

## Abbreviations

The following abbreviations are used in this manuscript:

AOI	area of interest
Qe	quiet eye
Avg fx	average fixation duration
H	head area of interest
T	torso area of interest
TA	throwing arm area of interest
TF	throwing forearm area of interest
B	ball area of interest
NTH	non-throwing hand (including arm and forearm) area of interest
L	legs (including thigh and shank of right and left legs) area of interest
O	other area of interest
ET	mobile eye tracer
MD	mean difference
rb	rank biserial correlation
U	Mann–Whitney U statistic
Z	z score
IHF	International Handball Federation
ERP	event-related potential
